# Is neurally adjusted ventilatory assist feasible during anesthesia?

**DOI:** 10.1186/cc13460

**Published:** 2014-03-17

**Authors:** F Campoccia Jalde, P Sackey, P Radell, M Wallin

**Affiliations:** 1 Karolinska University Hospital Solna, Sweden

## Introduction

Neurally adjusted ventilatory assist (NAVA) has so far been used in minimally sedated intensive care patients. NAVA has not been applied in patients in the operation room. The effect of different sedatives/anesthetics on the electrical activity of the diaphragm (Edi) has not so far been studied. The aim of our study was to compare the effect of sevoflurane and propofol on the Edi signal and breathing pattern during sedation and anesthesia and also in combination with remifentanil.

## Methods

A randomized cross-over study comparing sevoflurane and propofol sedation and anesthesia in 10 juvenile pigs. Remifentanil 0.1 μg/kg/minute was added after a period of anesthetic agent administration. The animals were ventilated with NAVA with fixed level throughout the study. Respiratory variables were measured for the last 5 minutes of each 15-minute exposure.

## Results

The Edi signal and spontaneous breathing were preserved with both anesthetics. The breathing variability, expressed as the coefficient of variation (SD/mean) of the tidal volume (CVvt), was high with both drugs. CVvt was greater during with propofol than with sevoflurane (CVvt 32 vs. 18% during sedation and CVvt 23 vs. 14% during anesthesia). The frequency of sighs was higher with propofol both during sedation (29 vs. 12 sighs/hour) and anesthesia (21 vs. 1 sighs/hour) than with sevoflurane.

## Conclusion

NAVA can be applied during propofol and sevoflurane anesthesia in pigs, with well-preserved Edi and spontaneous breathing. The natural variability is maintained with NAVA even during anesthesia. In contrast to sevoflurane, propofol sedation and anesthesia is associated with a high frequency of sighs and post-sigh apneas, probably due to a centrally induced mechanism. Our data warrant studies of NAVA in humans undergoing anesthesia and surgery when neuromuscular blockade is not required.

